# Nursing education reform in South Africa – lessons from a policy analysis study

**DOI:** 10.3402/gha.v7.26401

**Published:** 2014-12-22

**Authors:** Duane Blaauw, Prudence Ditlopo, Laetitia C. Rispel

**Affiliations:** Centre for Health Policy & Medical Research Council Health Policy Research Group, School of Public Health, Faculty of Health Sciences, University of the Witwatersrand, Johannesburg, South Africa

**Keywords:** nursing, nursing education, professionalisation, policy analysis, education reform, South Africa

## Abstract

**Background:**

Nursing education reform is identified as an important strategy for enhancing health workforce performance, and thereby improving the functioning of health systems. Globally, a predominant trend in such reform is towards greater professionalisation and university-based education. Related nursing education reform in South Africa culminated in a new Framework for Nursing Qualifications in 2013.

**Objective:**

We undertook a policy analysis study of the development of the new Nursing Qualifications Framework in South Africa.

**Design:**

We used a policy analysis framework derived from Walt and Gilson that interrogated the context, content, actors, and processes of policy development and implementation. Following informed consent, in-depth interviews were conducted with 28 key informants from national and provincial government; the South African Nursing Council; the national nursing association; nursing academics, managers, and educators; and other nursing organisations. The interviews were complemented with a review of relevant legislation and policy documents. Documents and interview transcripts were coded thematically using Atlas-ti software.

**Results:**

The revision of nursing qualifications was part of the post-apartheid transformation of nursing, but was also influenced by changes in the education sector. The policy process took more than 10 years to complete and the final Regulations were promulgated in 2013. The two most important changes are the requirement for a baccalaureate degree to qualify as a professional nurse and abolishing the enrolled nurse with 2 years training in favour of a staff nurse with a 3-year college diploma. Respondents criticised slow progress, weak governance by the Nursing Council and the Department of Health, limited planning for implementation, and the inappropriateness of the proposals for South Africa.

**Conclusions:**

The study found significant weaknesses in the policy capacity of the main institutions responsible for the leadership and governance of nursing in South Africa, which will need to be addressed if important nursing education reforms are to be realised.

There is global recognition of the urgent need to scale up educational programmes for the production of more health professionals in order to address patient and population health priorities and improve health system performance (1–4). Around the world, nurses are the largest category of health care providers (5), and play a vital role in health sector reform initiatives (6). Given the centrality of nurses in the health care system, changes in the production, scope of practice, and education of the nurse workforce are essential strategies for effecting improvements in the functioning and impact of health care systems (1).

Globally, the predominant trend in nursing education over the last century has been towards greater professionalisation through the lengthening of training periods, and the shift from a hospital-based apprenticeship model to professional education in institutions of higher learning (7, 8). Nevertheless, many countries still allow multiple pathways to registration as a professional nurse, generally with 3 or 4 years of higher education, and obtaining either a nursing college diploma or a university degree (9).

In recent years, an important development in the professionalisation of nursing education has been the call for a baccalaureate degree as the minimum requirement for entry to the profession (10). Proponents have argued that a university degree is required to cope with the increasing complexity of contemporary nursing practice resulting from a combination of factors including changes in patient and disease profiles, advances in medical and information technology, the shift to evidence-based practice, the need for life-long professional development, the challenges of working in health care teams, and the demands of ongoing health system reforms (3, 11–13). However, the shift to a baccalaureate degree as entry to nursing practice is also influenced by the desire to enhance the professional status of nurses, attract high-quality students, escape medical domination, and allow for more autonomous nursing practice (14, 15).

In South Africa, increasing professionalisation and a shift to university education have been important features of the reform of nursing education (16, 17). The first nursing school was established in 1877, following the standard hospital apprenticeship model of the time, and despite the efforts of its founder, it was placed under the jurisdiction of the Medical Council, instead of the Department of Education (18). The establishment of the South African Nursing Council (SANC) in 1944 at least wrested control of nursing education from the medical profession (19). However, a 3-year diploma at a hospital-based nursing college remained the only pathway to qualifying as a registered nurse (20). The first university nursing degree programmes in the country were introduced in 1956 but uptake remained relatively small (21). A more significant policy shift occurred in 1986 when all nursing colleges were required to become affiliated with university-based nursing departments, which placed them officially within the higher education system (16). At the same time, a new comprehensive 4-year curriculum (including general nursing, midwifery, community nursing, and psychiatric nursing) was introduced for the training of professional nurses in South Africa, which could be completed through a nursing college diploma or a university degree (22).

Since South Africa's democracy in 1994, there has been a renewed focus on nursing education as part of the post-apartheid transformation of both the health and higher education sectors (23). The nursing education policy reforms have included the rationalisation of nurse training institutions, changing the scope of practice of nurses, and revising nursing qualifications (22–25). The revision of nursing qualifications has been driven by changes within the profession and the imperative to align nursing qualifications with the new National Qualifications Framework (NQF) – a comprehensive system for the classification and articulation of qualifications in the country (26). A key recommendation of the new nursing qualifications proposals is that registration as a professional nurse will require completion of a baccalaureate degree in nursing, rather than a nursing college diploma.

In light of South Africa's health care reforms to achieve universal coverage (27) and the importance of nursing education in the preparation of nurses for their roles in leading and implementing these reforms, this study analysed the context, content, actors, and process of the development of the new Framework for Nursing Qualifications in South Africa.

## Methods

### Conceptual framework

Contemporary policy analysis is based on the premise that policy-making is inherently political in nature and seeks to provide a systematic method for analysing policy processes and the factors that influence them (28). Although a number of frameworks and tools have been developed for health policy analysis (28–31), this study used an analytical framework derived from Walt and Gilson (30) that interrogates the context, process, content, and actors of policy development and implementation. Context refers to the broader situational and structural factors influencing the reform. The process analysis investigates the way in which policies are identified, formulated, and implemented; the timing of events; and the strategies used at each stage. The analysis of content focuses on the nature and details of the policy proposals. The study of actors is concerned with the key stakeholders involved in developing and implementing the reforms, as well as their differing roles, values, interests, and influence (30).

### Policy of focus

Although there have been a number of reforms in nursing education in South Africa since 1994 (23), this study analysed the context, process, content, and actors involved in the development of the new Nursing Qualifications Framework in South Africa. Essentially, this policy reform was concerned with defining the different categories of nurses in South Africa, specifying the minimum qualifications required for each category of nurse, and describing the articulation between them.

The study reported in this paper was part of a larger project to examine the dynamics, strengths, and weaknesses of nursing policy-making in South Africa (32). It constitutes one of four policies of focus identified at a broad consultative workshop held with key nursing stakeholders (33).

### Study sites

The focus of this study was primarily on national policy processes. Although actors at the national level may drive nursing policy development, the responsibility for implementation is with provincial managers and the ultimate impact of policies is on frontline nurses. Therefore, the project collected data from all three levels. Sub-national data collection focused on four provinces: Gauteng, Eastern Cape, Free State, and Western Cape.

### Data collection and study participants

The overall study design was an in-depth, qualitative, case study of the new Nursing Qualifications Framework in South Africa. Case study methodology allows for a detailed exploration of the complex dynamics and relationships within a real-life context (34). The researchers also sought to maximise participation and policy engagement with policy actors as part of the research process.

The research team reviewed relevant legislation, regulations, government and nursing council documents, and inputs made by nursing education stakeholder groups in order to understand the context and content of the new Nursing Qualifications Framework.

The research team conducted interviews with 28 purposively selected key informants, drawn from six categories: national and provincial government officials (*n*=14), nursing managers from the private sector and non-governmental organisations (*n*=5), nursing academics in colleges and universities (*n*=6), and representatives from the SANC and other nursing organisations (*n*=3).

Semi-structured face-to-face interviews were conducted in English after obtaining written informed consent. If the key informant (KI) agreed, the interviews were recorded to improve accuracy and to support more detailed analysis. The interviewer also kept detailed notes of each interview. The issues covered with each KI included: aims of the policy; policy design and content; contextual factors influencing policy development; important milestones in the policy process; the roles, interests, and influence of different actors; motivation for the strategies adopted; factors influencing the policy process; and informants’ interpretation of the success or failure of the policy initiative.

### Data analysis

Documents, interview transcripts, and written notes were analysed using standard approaches to qualitative data analysis (35, 36). The analytical themes were derived from the specific research objectives, the conceptual framework of the study, and the data. The Atlas-ti software programme was used to support qualitative data analysis. The analysis of process included the careful documentation of key milestones, content development, and strategic shifts. The actor analysis included a formal stakeholder analysis (31).

### Ethical considerations

National and international ethical standards were followed throughout the research. The University of the Witwatersrand Human Research Ethics Committee (Medical), and the relevant provincial health authorities granted permission to conduct the study.

Respondents were provided with a study information sheet explaining the purpose of the study and the terms of their consent. Those who agreed to be interviewed were asked to sign the study consent form. Separate consent was also obtained for any recording of interviews. Personal identifiers were removed from all transcripts and results.

## Results

The context, process, content, and actors relevant to the development of the new Nursing Qualifications Framework are described briefly, followed by respondents’ main criticisms of the policy process.

### Context

Table 1 presents a timeline of key events in the development of the new Nursing Qualifications Framework, as well as significant contextual developments in the broader nursing and education sectors.

**Table 1:** Timeline of key events 1994–2013: new Nursing Qualifications Framework (see text for abbreviations).

Year	General Health/Nursing Sector	Nursing Qualification Framework	Educational Sector
1978	Nursing Act no. 50		
1985		4 year comprehensive course introduced (R425)	
1995			SAQA Act no. 58 establishes NQF with 8 bands
1997	White Paper for the Transformation of the Health System		White Paper for the Transformation of Higher Education Higher Education Act no. 101
1998			Further Education and Training Act no. 98
1999	Rationalisation (closing) of nursing colleges National Summit on Nursing, Kopanong		
2000	Pick report on Human Resources for Health		
2001		Nursing SGB established	General and Further Education and Training Quality Assurance Act no. 58
2003	National Health Act no. 61	Nursing SGB releases draft NQF qualifications for public comment	
2004		Nursing SGB finalises qualifications for registration on NQF	
2005	Nursing Act no. 33		
2006	New DDG appointed for Human Resources in NDOH Human Resources Strategic Framework		Further Education and Training Colleges Act no. 16
2007	Sections of Nursing Act promulgated but awaiting required Regulations	SANC revises qualifications for registration on NQF	Review of SAQA and NQF completed New Higher Education Qualifications Framework (HEQF) with 10 bands to be implemented by 1 January 2009
2008	Nursing Strategy		National Qualifications Framework Act no. 67
2009	New Minister of Health, Dr Motsoaledi, appointed	SANC sets deadline of 30 June 2010 for phasing out of legacy qualifications	
2010	New Director-General, Ms Matsoso, appointed	Nursing education stakeholders develop proposals for HEQF qualifications Legacy qualifications extended to 30 June 2012 NDOH commissions independent audit of all training institutions but report not released	
2011	Nursing Summit and Compact, April Revised Human Resources for Health Plan, October Ministerial Task Team on nurse education and training established Acting Chief Nurse appointed in NDoH. Official post advertised	Qualifications framework debated at Nursing Summit Legacy qualifications extended to 30 June 2013 Draft Regulations released for public comment on 14 Dec 2011	
2012		Legacy qualifications extended to 30 June 2015	
2013		SANC task team finalised new qualifications to be submitted to SAQA 8 March: New qualification Regulations gazetted Strategic Plan for Nursing Education, Training and Practice released New programmes to be submitted by 30 Nov for accreditation by CHE and SANC	

**Fig. 1:**
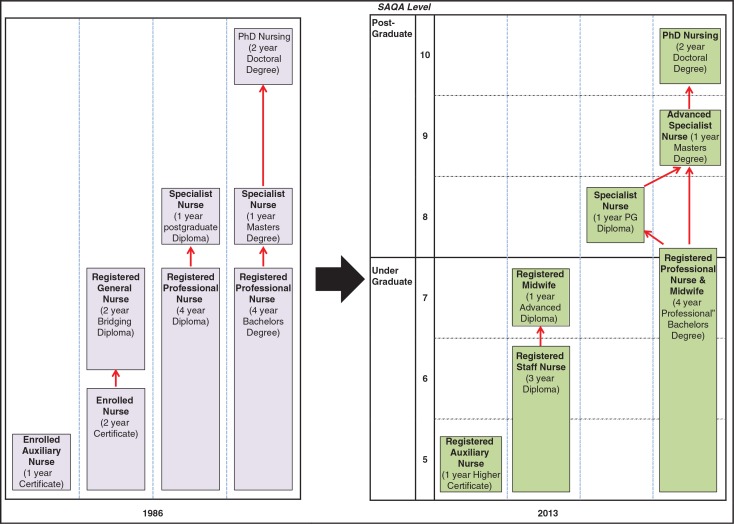
Changes in the Nursing Qualifications Framework.

KIs argued that the revision of nursing qualifications was an important part of the on-going transformation of nursing in South Africa, and not only a response to changes in the higher education sector (Table 1).

The development of the new qualifications is about updating, modernising the practice of nursing. [KI 2, National Department of Health]

For the profession we need a professional nurse that's a graduate nurse that is in line with the rest of the world and the new qualifications would give us that. [KI 12, Nursing Academic]

The post-apartheid transformation of nursing legislation culminated in the passing of a new Nursing Act in 2005. Although the Act was promulgated in 2007, many provisions of the act required further specification in Regulations. KIs pointed out that an important contextual factor was the development of draft Regulations outlining the Scopes of Practice of different categories of nurses and the qualifications required for each category. Nursing stakeholders were dissatisfied with the Scope of Practice Regulations and qualifications inherited from the previous Nursing Act of 1978 and had strongly advocated for an overhaul of these provisions. KIs explained that the main motivations for education reform included: insufficient distinction in the roles of different nursing categories, the need to address changing disease patterns and health system priorities, and the need to reflect the more independent practice of contemporary nurses. Others saw it as an opportunity to review the 4-year comprehensive nursing course introduced in 1986 which paradoxically had been criticised both for excessive training in all four areas of care, and for inadequate preparation of graduates for competent practice in all of these areas, particularly midwifery.

We have got a challenge of high maternal and child mortality. When you ask people what could be the cause of that, they will then begin to look at this 4-year programme and ask whether it produces competent midwives or not? [KI 18, Nursing Association]

The post-apartheid era also brought broader challenges for nursing that needed to be considered in the revision of the nursing qualifications. These included increased service demands, easier international migration, staff shortages, decline in the image and status of the profession, difficulties in attracting good recruits, an ageing workforce, and generally low staff morale (22, 24).

Another critical contextual factor was the development of a new NQF by the Department of Education. Prior to 1995, the SANC was the main regulator of nursing training and qualifications but the introduction of the South African Qualifications Authority (SAQA) and the new NQF meant that nursing qualifications now had to comply with broader Department of Education policy.

A 2007 review of SAQA and the NQF resulted in a significant revision of the NQF to develop the new Higher Education Qualifications Framework (HEQF). The revised NQF consists of ten levels, instead of the previous eight, with each level providing a broad indication of the types of learning outcomes and assessment criteria that are appropriate to a qualification at that level (26). KIs explained that the new nursing qualifications had to keep up with this changing national context.

### Process

The timeline in Table 1 shows that the new Nursing Qualifications Framework policy process has taken more than 10 years to complete. The related work on the Scope of Practice Regulations has taken even longer since the initiative began in 1997 and was not yet finalised by 2013.

The reform of nursing education and training can be divided into two main phases.

The first phase of the reform process occurred between 2001 and 2009 and was primarily concerned with aligning existing nursing qualifications with the NQF. These were the so-called legacy qualifications inherited in 1994 for the training of enrolled nursing auxiliaries (1 year training), enrolled nurses (2 years of training), and diploma- or degree-qualified professional nurses with 4 years of training. SAQA had defined specific processes for this that involved the establishment of a Nursing Standards Generating Body (SGB). The Nursing SGB produced various draft qualifications between 2002 and 2004 but the process stalled because of lack of resources. A further difficulty was the change from the NQF to the HEQF, which meant that the work done so far had to be revised. The SANC took over finalising the registration of the legacy qualifications with SAQA, eventually completing the task in 2009.

The second phase of reform from 2008 to 2013 dealt with the development of a completely new Nursing Qualifications Framework, also aligned with the HEQF which was meant to be led by SANC. Timeframes were not adhered to, and despite an initial deadline of 30 June 2010 being set for the phasing out of the legacy qualifications, the process has continually been extended. The new deadline, at the time of writing, is the 30 June 2015.

Although the involvement of nurse educator groups resulted in some increased momentum, respondents argued that progress has been sporadic and the process laboured. In April 2011, a national Nursing Summit was held where the following statement regarding the new Nursing Qualifications Framework was made (37):

We call upon SANC in collaboration with the National Department of Health, CHE [Council for Higher Education] and SAQA to fast-track the processing and implementation of the new Nursing Qualifications’ Framework and appropriate transitional arrangements.

Following the summit, the Minister of Health appointed a Ministerial Task Team on Nursing Education and Training which finally provided the impetus to complete this phase of the policy process. Regulations for the new nursing academic qualifications were released for public comment in December 2011 and finally promulgated in March 2013. The Minister of Health also released the new Strategic Plan for Nursing Education, Training, and Practice in March 2013 (25). However, both SAQA and the Council for Higher Education (CHE) have to approve the new qualifications and teaching programmes prior to their implementation in 2015.

### Content

Figure 1 provides a graphic representation comparing the pre-1994 qualifications and the final new Qualifications Framework as outlined in the 2013 Regulations. Since 1985 the main nursing qualifications recognised in South Africa were as follows (22, 23):

1. Enrolled nursing auxiliary: 1-year certificate at a training hospital;
2. Enrolled nurse: 2-year certificate at nursing college;
3. Professional nurse: 4-year diploma at nursing college or 4-year baccalaureate degree at university.

**Fig. 2:**
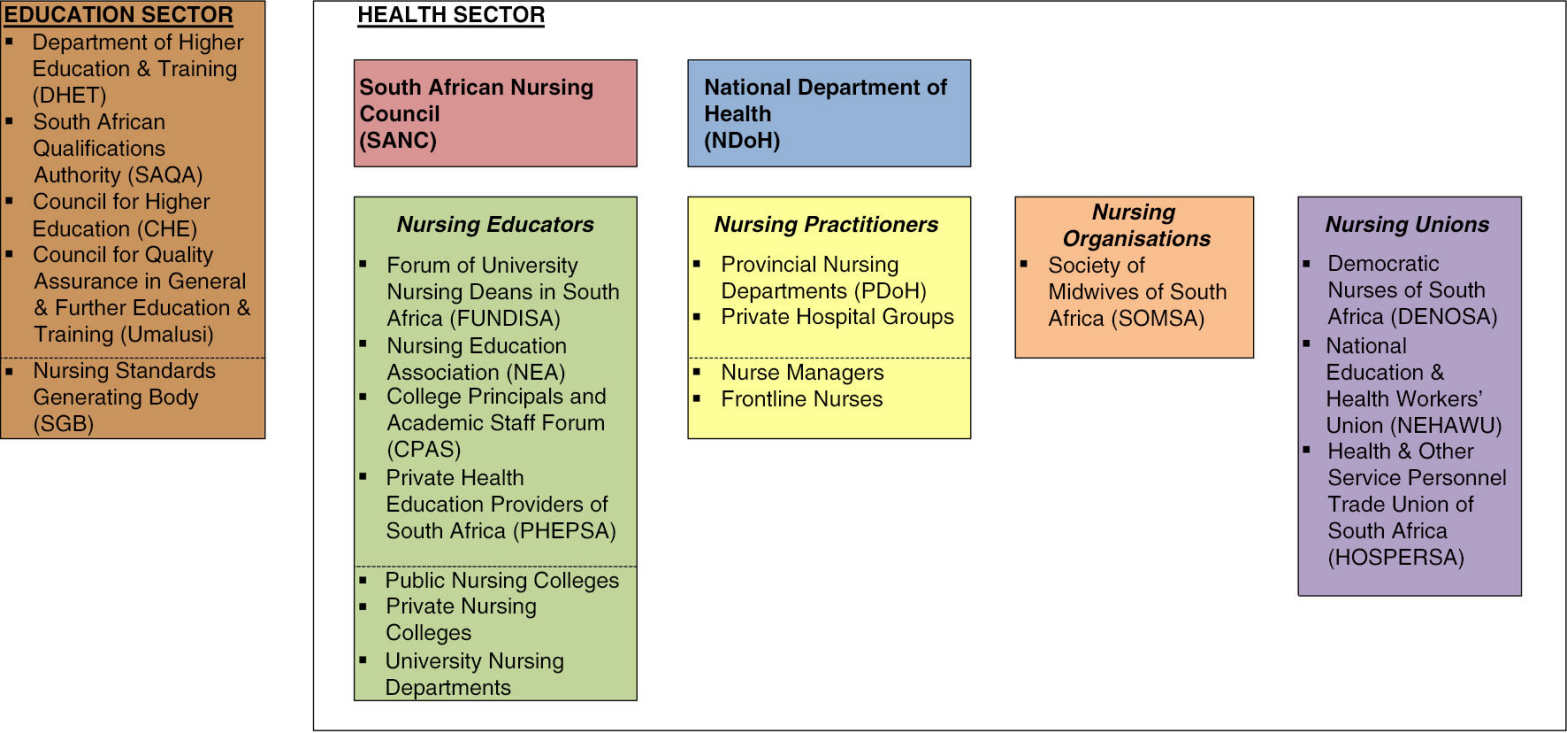
Mapping of key actors involved in the Nursing Qualifications Framework policy process.

A bridging programme also made it possible for enrolled nurses to complete a 2-year diploma at a nursing college and qualify as a registered general nurse. Specialised nurses completed either a postgraduate diploma or master's degree, and doctoral nursing programmes were available at universities.

The new Qualifications Framework includes two fundamental changes (Fig. 1). First, registration as a professional nurse will now require completion of a 4-year baccalaureate degree at a university. This is in keeping with trends in high-income countries, and satisfied some nursing educators concerned about the declining professional status of nurses in South Africa. The bridging course has also been withdrawn so there is now only one pathway to becoming a professional nurse instead of three. Second, the enrolled nurse has been replaced by a staff nurse with a 3-year college diploma at HEQF level 6. This was mainly because a 2-year diploma is not permissible on the revised HEQF. But respondents also argued that a reconfigured 3-year staff nurse qualification would be a significant step towards meeting South Africa's basic nursing needs.

I understand that the needs of the country are about an expanded scope of practice for an enrolled nurse. [KI 26, Nursing Academic]

The scope of practice of the new staff nurse has been broadened so that they will be able to work more independently and provide basic nursing care to uncomplicated patients in all settings.

### Actors

The key actors involved in the nursing qualifications policy process are represented in Fig. 2.

Overall governance and leadership of the nursing sector is shared between the SANC and the Human Resources Division of the National Department of Health (NDoH). Other important actors from the health sector included nursing educators, nursing practitioners, nursing organisations and the national nursing association, and health sector unions. Nursing educators include individual training institutions, as well as educator groupings such as the Forum of University Nursing Deans in South Africa (FUNDISA), the Nurse Educators Association (NEA), the College Principals and Academic Staff (CPAS) forum, and the Private Health Education Providers of South Africa (PHEPSA) (Fig. 2) representing university, nursing college, and private educators. A significant development highlighted by this policy case study is the increasing role of actors from the education sector, including the Department of Higher Education and Training, SAQA, and the CHE (Fig. 2), in nursing education and training.

Various actors have played a central role at different stages of the process. Phase 1 of the process was initially driven by the Nursing SGB, a sub-structure of SAQA, intended to ensure broader representation in the development of national qualifications.

In 2001, there was the Nursing SGB which played a great role in developing the Nursing Standards. The very new qualifications that we are talking about now, they were given birth to by the Nursing SGB. [KI 16, Nursing Educator.]

Not surprisingly, the SGB came into conflict with SANC, which had previously been the only actor responsible for the regulation and development of nursing qualifications. Ultimately, the SANC reasserted its role and the Nursing SGB was abandoned.

Nursing educators dominated the phase 2 process. The so-called Nursing Education Stakeholders (NES), a group representing all nurse educator organisations, developed the initial proposals and advocated and lobbied extensively for the new Nursing Qualifications Framework. A workshop held by the NES in February 2010 defined the subsequent development of the debate and they played a prominent role in the presentations and discussions on the Qualifications Framework at the Nursing Summit in April 2011. Despite initial differences within the NES group, particularly from PHEPSA and CPAS, the NEA-FUNDISA proposals came to be accepted and disseminated.

One of the success stories that FUNDISA has played a critical part in is coming up with a Nursing Qualification Framework that SANC has now adopted. [KI 26, Nursing Academic]

The finalisation of the Regulations was subsequently taken forward by the Ministerial Task Team on Nursing Education and Training. The influence of the NDoH, particularly the Human Resource Division, has been uneven. At an earlier stage some officials appeared uncomfortable with the shift to greater professionalisation through the baccalaureate degree.

Our Minister at one point said, ‘the way that South Africa is going with its academic qualifications, very soon we will be saying that Agricultural Extension Officers must train at university, and the country will be without food because how can someone who comes from the university know how to farm, how to till the soil.’ That is the way it is. [KI 39, National Department of Health]

Nevertheless, the Minister of Health signed the final Regulations, including the requirement of a baccalaureate degree for professional nurses.

### Stakeholder criticisms of the policy process

Table 2 summarises key informants’ main criticisms of the nursing qualifications policy process.

**Table 2:** Key criticisms of new nursing qualifications policy process

Context issues	Context determined by education sector rather than nursing. Process unable to keep up with changing context.
Process issues	Policy progress took too long. Limited planning for actual implementation.
Content issues	Nursing colleges have to be registered as higher education institutions. Limited evidence base for proposed revisions. Uncertainty whether proposals match health needs of the country. Debates about direct-entry midwifery.
Actor issues	Weak leadership and governance by SANC and NDoH. Domination by nurse educators with limited involvement of employers and frontline nurses. Limited real engagement with NDoH.

With regard to contextual factors, SANC and nursing stakeholders have grappled with the introduction of the Department of Education into the regulation of nursing education.

I mean the Council has always had a Qualifications Framework, but it belonged to the Council and now it had to start merging with the education system of the country. [KI 9, Nursing Educator]

Some informants also highlighted the difficulties of dealing with the ongoing changes in the education sector.

We were busy writing those nursing qualifications of the previous Council and then they got them complete and ready to implement and then they realised the NQF had changed. [KI 25, Nursing Manager]

Not surprisingly, in terms of process, a major concern has been the very slow pace of policy development, particularly in relation to producing the enabling Regulations required by the new Nursing Act.

Although the ground work has been done, the implementation is completely at a standstill. There is absolutely no implementation in any aspect of the Nursing Act or any of those draft Regulations that are there. [KI 24, Former SANC Member]

Some respondents were also concerned about the limited planning for the implementation of the new qualifications, which will have a significant impact on nursing education, workforce planning, and existing nurses. Educational institutions still need to develop and implement new curricula and training programmes, for example. A more fundamental problem is that it is envisaged that the staff nurse diploma will be provided by nursing colleges, but HEQF level 6 is in the Higher Education band and most public and private nursing colleges are not currently registered as higher educational institutions – and the process for changing this is neither quick nor simple.

A few interviewees questioned the evidence-base for the content of the policy recommendations:

So for me I am not very sure if enough research was done in terms of making a point that the new qualifications are actually tailor-made to suit the current demands of the country. [KI 14, Manager, Free State Department of Health]

This echoed concerns about whether the current educational proposals meet South Africa's health needs. Some respondents were of the opinion that the tendency towards professionalisation reflected a preoccupation with international comparability, status, and earning potential rather than local health priorities.

The case study revealed a number of problems with key policy actors. The policy process stalled between SANC and the NDoH, but it was unclear which organisation was primarily responsible for the delay. SANC blamed the NDoH:

And why they [Regulations] were not published, I think there were challenges in the Department of Health. [KI 24, Former SANC Member]

At the same time, the NDoH blamed the SANC:

It's a matter of just passing them I think it has been more the Council problems that have stood in the way for those Regulations. [KI 1, National Department of Health]

Other interviewees highlighted weaknesses of both of these key actors in relation to governance of the nursing sector. They were blamed for the poor coordination between them and their failure to provide leadership to complete the policy process:

I really think that it seems as if one body waits for the other instead of there being somebody that drives the process and then takes it through to completion. [KI 23, Nursing Manager]

With regard to SANC, problems with the appointment of a registrar, internal divisions, weak administration, changes in the SANC board, and the subordinate role of the new Council in relation to the Minister were blamed for the slow progress:

It's a dysfunctional body I have to say. The sad thing is that it has been going on for years; it's not just this Council. When I was on Council both terms were relatively dysfunctional. [KI 7, Manager, Gauteng Department of Health]

The NDoH also came in for criticism due to their limited capacity in driving the policy reform processes, and the fact that there was no dedicated unit or senior manager responsible for nursing issues. Some respondents felt that the NDoH had not been sufficiently involved in the planning of the new nursing qualifications.

Finally, other actors complained about being side-lined in the qualification framework process, and argued that there had been inadequate consultation on an issue that would have significant long-term consequences for all nurses in South Africa:

In that discussion the unions and the practitioners have not been involved. It's really been agreed by the nursing council and universities. [KI 19, Nursing Association]

## Discussion

This is one of the first policy analysis studies in South Africa concentrating exclusively on policy development and implementation in the nursing sector. This case study of the development of a new Nursing Qualifications Framework indicates significant weaknesses in the political and technical capacity of the main institutions that are supposed to provide leadership and governance for policy-making in the nursing sector in South Africa.

The final Nursing Qualifications Framework developed for South Africa, in proposing both a baccalaureate degree for professional nurses and new general staff nurses, is an interesting compromise that follows the global trend towards more professionalisation, but also attempts to address nursing shortages and the significant deficit in basic nursing care in the country.

A few high-income countries, including Belgium, Italy, Spain, Norway, Canada, the United Kingdom, Australia, and New Zealand, have recently made the baccalaureate degree a requirement for registration (8, 38–40). Similar policies are being considered in other high-income countries. In the United States, for example, the American Nursing Association has officially supported baccalaureate entry to practice since 1965 (41, 42), and the recent influential Institute of Medicine monograph on the future of nursing recommended that baccalaureate nurses should make up 80% of the total nursing workforce by 2020 (3). However, discourses regarding nursing education reform in low- and middle-income countries are different. The emphasis is more on educational changes required to address the global shortage, maldistribution, retention, skills mix, scope of practice, and performance of nurses so as to strengthen health systems, scale up priority health interventions and achieve universal coverage (43–46). For example, the recent World Health Organization (WHO) report on global standards for the initial education of professional nurses and midwives makes reference to the global shift towards university-based nursing education but identifies this as a goal for the future (47). Nevertheless, baccalaureate entry to practice reforms have also been implemented in a few middle-income countries, such as the Philippines and Brazil, and are being considered in others, including Mexico, India, and Jordan (9, 48).

The influence of nursing educators in the South African policy process contributed to acceptance of the baccalaureate degree requirement for professional nurses, despite initial concerns from NDoH officials that this was not appropriate or cost-effective for South Africa (23). Although it is frequently stated that university-trained nurses are more capable of dealing with the demands of contemporary nursing, and a number of countries have made this a requirement for practice, the empirical evidence comparing the competence of degree- and diploma-trained professional nurses is more mixed (49–53). In addition, some commentators have questioned whether the nursing degree requirements recently introduced in Canada and Australia are delivering everything they promised (39, 40).

There are some limitations of this policy analysis case study. Although many of the key stakeholders involved in the policy process were interviewed, it was sometimes difficult to cover all of the issues within the time constraints of a single interview. Another difficulty was KI recall bias and the reliance on a retrospective report of events, some of which happened many years previously. Where possible, we corroborated accounts between different respondents and compared the interviews with the documentary evidence. A further limitation is that the analysis focuses mainly on the policy development phase – policy implementation has barely begun and is likely to present additional challenges. Nonetheless, the policy analysis approach of this case study, and its focus on broader systems of governance and leadership within nursing, are unusual in the nursing literature. Most of the available international literature linking nursing and policy-making is concerned with the absence of nurses in broader health policy processes (54–58). There is also a fairly large body of work on the professional regulation of nurses (59, 60), but this literature has a mostly technical focus and has seldom drawn explicitly on approaches from the political and policy sciences.

Reports outlining the most urgent priorities for nursing education reform are frequent (3, 61–63). However, more analysis is required of the leadership, institutional capacity, and policy processes required to implement such reforms in different countries. The nursing education literature has also focused mostly on the technical aspects of nursing education reform – changes in qualifications, competencies, curricula, and pedagogy, for example – but commented little on the politics and processes of nursing education reform. This paper identifies considerable failings in the processes of policy development for policies within the nursing sphere in South Africa. The analysis points to inadequate nursing policy-making expertise and leadership within SANC and the NDoH, as well as poor coordination between them, that is likely to undermine future policy development and implementation in nursing education. Certain actors, such as nursing educators and unions, appear to have some policy competence, but are unable to overcome the weaknesses within the SANC and the NDoH, who bear ultimate responsibility for nursing policy development and implementation.

A few other authors have commented on the capacities and processes required to support reform in nursing education, at the macro and meso levels. For example, the WHO guidelines on transformative scale-up of health professional education emphasise the importance of national leadership, governance, and planning of health education reform (4). Spitzer and Perrenoud (64) analysed the organisational capacities required to support nursing education reforms in Western Europe aimed at transferring nursing education into universities. Lastly, recent studies have highlighted the institutional weaknesses of many nursing and midwifery regulatory bodies in central, east, and southern Africa and their failure to enact the important educational and regulatory reforms needed to cope with new health system priorities (65, 66). Clearly, more research on this topic is necessary.

It is encouraging that nursing stakeholders eventually finalised a new Framework for Nursing Qualifications in South Africa and produced the Regulations required by the Nursing Act of 2005. Also, the Strategic Plan for Nursing Education, Training, and Practice released by the NDoH in March 2013 (25) thoroughly summarises the current challenges facing nurses in South Africa, provides a relatively comprehensive reform strategy, and details an implementation plan for achieving it (25). However, one weakness of the new strategic plan is that it fails to interrogate the limited success of previous policy initiatives in the nursing sector, including the previous Nursing Strategy of 2008 (24). Policy analysis, such as that presented in this paper, can provide useful insights to strengthen future policy development and implementation. The analysis presented in this paper suggests that the new plan is unlikely to be successful without significant interventions to improve the policy and implementation capacity of SANC and the human resource division of the NDoH. Although South Africa's first Chief Nursing Officer was appointed in January 2014, implementation of the plan is already falling behind its own strategic objectives and planned timelines. Furthermore, the new strategic plan process has been a missed opportunity to tackle the systemic and structural weaknesses in the policy machinery required to drive nursing reform in South Africa.

## Conclusions

The performance and health outcomes of the South African health system are disappointing given the country's level of economic development. There is renewed commitment and energy to addressing the problem and a number of health system reforms are currently underway (27, 67). However, a successful turnaround depends on improving the performance of nurses, the largest category of health providers. This, in turn, depends on the effective implementation of proposed changes in the education, training, and practice of nurses. This study suggests that there is sufficient expertise in the country to analyse the problems and make recommendations for educational reform. However, the study also shows that there is a fundamental and longstanding crisis in the institutional governance and leadership of the nursing sector in the country. The policy capacity of key institutions requires urgent strengthening if these important nursing education reforms are to be realised.
